# Selective Internal Radiotherapy Alters the Profiles of Systemic Extracellular Vesicles in Hepatocellular Carcinoma

**DOI:** 10.3390/ijms241512512

**Published:** 2023-08-07

**Authors:** Severin Gylstorff, Vanessa Wilke, Daniel Kraft, Jessica Bertrand, Maciej Pech, Florian Haag, Borna Relja

**Affiliations:** 1 Experimental Radiology, Department of Radiology and Nuclear Medicine, Otto-von-Guericke-University, 39120 Magdeburg, Germany; 2 Research Campus STIMULATE, Otto-von-Guericke University, 39120 Magdeburg, Germany; 3 Translational and Experimental Trauma Research, Department of Trauma, Hand, Plastic and Reconstructive Surgery, University Ulm, 89081 Ulm, Germany; 4 Department of Orthopaedic Surgery, Otto-von-Guericke-University, 39120 Magdeburg, Germany; 5 Department of Radiology and Nuclear Medicine, University Medical Center Mannheim, Heidelberg University, 68167 Mannheim, Germany

**Keywords:** cancer, HCC, EV, biomarker, diagnosis, prognosis

## Abstract

Incidence of hepatocellular carcinoma (HCC) is increasing globally. Radioembolization (RE)/selective internal radiotherapy (SIRT) is a promising treatment for inoperable HCC. RE triggers an immune response, involving extracellular vesicles (EVs) which are crucial for cell communication and tumor development. This study explores EV immune profiles and origins in patients with inoperable HCC before and after SIRT/RE. Blood samples from 50 HCC-patients treated with SIRT/RE were collected before and after therapy to determine cytokines and isolate EVs using size exclusion chromatography. The dynamic range and EV quality required for detecting variations in surface markers were assessed. Thirty-seven EV surface markers were analyzed using flow cytometry and correlated with clinical parameters. Several immunological markers (CD4, CD2, CD40, CD45, CD49e, CD69, CD209-EVs) were present in the circulation of HCC patients. These markers positively correlated with therapy response and survival. Conversely, B cell CD20, endothelial cell CD146, platelet CD49e, and CD41b EV markers negatively correlated with 60-day survival. Elevated levels of IL-6 and IL-8 before therapy correlated negatively with patient survival, coinciding with a positive correlation with CD20-positive EVs. Plasma EVs from HCC patients exhibit immunological, cancer, and coagulation markers, including potential biomarkers (CD4, CD20, CD49e, CD146). These may enhance our understanding of cancer biology and facilitate SIRT therapy monitoring.

## 1. Introduction

Hepatocellular carcinoma (HCC) is showing rising incidence in the most western countries [1]. It is the sixth most common cancer, and the third leading cause of cancer-related death worldwide [1]. Major risk factors for the development of HCC are chronic alcohol consumption and chronic hepatitis B and C, as well as non-alcoholic fatty liver disease [2]. According to international recommendations, the staging of HCC is crucial when choosing an appropriate treatment strategy. Patients who possess a well-functioning liver and a small tumor size have a more favorable prognosis compared to those with impaired liver capacity and a larger tumor. In cases of preserved liver function, a resection of the tumor can be performed [3,4]. Unfortunately, a significant majority (over 70%) of patients diagnosed with HCC have already reached an advanced stage at which surgical resection or liver transplantation is no longer a viable option [5]. Selective internal radiation therapy (SIRT) is considered a crucial treatment option for non-operable HCC, and plays a significant role in the management of these cases [3,5]. During SIRT, a radioactive substance such as Yttrium-90 is administered directly into the bloodstream of the tumor through an arterial catheter. This targeted delivery of radiation causes necrosis within the tumor tissue, and it additionally promotes the infiltration of immune cells into the tumor [6]. Previous research has demonstrated a correlation between the activation of both local and systemic immune responses and the long-lasting effectiveness of SIRT in patients with HCC [6,7]. Extracellular vesicles (EVs) are involved in mediating immune responses and facilitate intercellular communication; thus, they may play an important role in immune cell activation, inflammation, and immune system regulation during tumor therapies [8]. EVs are small microparticles enclosed in a lipid bilayer, typically ranging from 30 to 10,000 nm. They can be released by various cell types, including cancer cells, and contain a cargo of molecules derived from their parent cells, such as functional microribonucleic acids (miRNA), messenger RNA (mRNA), deoxyribonucleic acids (DNA), and proteins [9,10]. Upon uptake by recipient cells, these molecules can exert their functional effects. EVs have emerged as novel biomarkers and potential therapeutic targets in the field of cancer diseases [11]. They are classified into three main categories: exosomes, microvesicles, and apoptotic bodies [10]. Different types of EVs can be distinguished, isolated, and categorized based on their surface antigens such as cluster of differentiation (CD)9, CD63, and CD81, which are commonly used to identify and characterize specific subpopulations of EVs [12,13,14]. Since circulating EVs are believed to play a crucial role in carcinogenesis and the tumor microenvironment, they may reflect distinct inflammatory mechanisms during tumor progression, and have the potential to serve as prognostic biomarkers in cancer patients [8,15]. Webber et al. demonstrated that the exosomes released by cancer cells can transfer information to normal stromal fibroblasts, eliciting a cellular response. Their findings revealed that the transforming growth factor beta (TGFβ)1 on their surface induced the differentiation of fibroblasts into tumor-promoting myofibroblasts, which exhibit pro-angiogenetic properties [16,17]. Additionally, the promising diagnostic potential of circulating EVs in the context of hepatocellular, ovarian, and pancreatic carcinoma was shown [18,19]. Furthermore, the expression of surface antigens CD105 and CD146 on EVs showed a prognostic potential in patients suffering from metastatic colorectal cancer [20]. However, there is currently limited knowledge regarding circulating EVs specifically associated with SIRT in the treatment of HCC. It is reasonable to assume that clusters of EV patterns could potentially reflect distinct inflammatory mechanisms during tumor progression and SIRT in HCC patients. These EVs patterns may hold prognostic and diagnostic value, offering insights into the underlying processes and treatment response for HCC patients.

## 2. Results

### 2.1. Patient Characteristics

**Figure 1:**
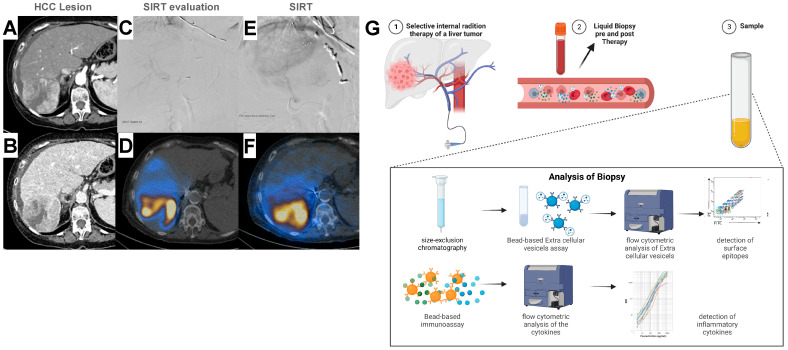
Procedure of selective internal radiotherapy (SIRT) in patients with hepatocellular carcinoma (HCC). (**A**,**B**) Computed tomography (CT) scan of an HCC lesion in the right liver lobe. Typically, early contrast media enhancement is shown in the arterial phase (**A**) combined with wash-out in the venous phase (**B**). (**C**) Application of 99mTc-MAA via an arterial catheter after isolation of arterial hepatic blood supply. (**D**) Detection of 99mTc-MAA using single-photon emission CT (SPECT). (**E**) Application of Yttrium-90 (YY90) via arterial catheter. (**F**) Control of 90Y enrichment using SPECT. (**G**) Schematic illustration of the experimental workflow (created with BioRender.com). HCC patients received a liver MRI according to clinical standards, and blood samples were taken to obtain plasma before (pre-T) and after (post-T) SIRT. Cytokine levels in plasma samples and extracellular vesicles (EVs) were isolated from plasma using an IZON isolation system, and analyzed using different experimental approaches.

The medical records of 50 patients, 10 women and 40 men who underwent SIRT were evaluated (henceforth referred to as the HCC cohort) (Figure 1). Within the observation period of 60 days, 9 out of 50 patients died. The patient characterization included age, sex, tumor volume, tumor fraction, and administered activity dose. Furthermore, clinical parameters such as creatinine, urea, uric acid, bilirubin, albumin, aspartate aminotransferase (AST), alkaline phosphatase (ALP), alanine aminotransferase (ALT), gamma-glutamyl transferase (GGT), quick, international normalized ratio (INR), partial thromboplastin time (PTT), thrombin time, and C-reactive protein (CRP) were recorded. The descriptive baseline characteristics of the HCC cohort are provided in Table 1.

### 2.2. Clinical Characteristics Associated with Treatment and Outcome

The clinical characteristics that were found to be significantly associated with SIRT are shown in Table 2. Analyses of cell counts pre-T versus post-T revealed significant changes in erythrocytes, thrombocytes, neutrophils, basophil granulocytes, and lymphocytes, as well as monocytes (*p* < 0.05, Table 2). The total number of leukocytes did not change.

The HCC cohort was divided into four different study groups. Survivors and non-survivors were compared before therapy and after therapy (Table 3). Furthermore, pre-T and post-T comparisons were made within the survivor group and non-survivors (Table 3). The median age of the HCC cohort survivors was 69 [range 62.75–79.00], the median age of the non-survivors was 70 [range 62.00–79.50]. In the survivor group, the median total liver volume was 1775 cm^3^ [range 1445–2469], the tumor volume was 163.50 cm^3^, and the median size of the tumor fraction was 5.4% [range 3.1–16.83]. While the tumor volume and tumor fraction did not substantially vary from the survivors, the median tumor volume in the non-survivor group was 2790 cm^3^ [range 1860–3332], significantly larger than that of the survivors (Table 3). In addition, there were significant changes in ALP levels in pre-T survivors compared to non-survivors (Table 3). Significant differences were observed post-T when CRP and AST levels were compared between survivors and non-survivors (Table 3). In contrast, in the pre-T vs. post-T survivor group, there were significant differences in the concentrations of urea, bilirubin, albumin, and GGT (Table 3). Further comparison between non-survivors pre-T and post-T showed significantly higher creatinine, hemoglobin, and albumin levels (Table 3). Significant differences were also found in AST and thrombin time pre-T and post-T (Table 3).

To detect systemic effects, the total number of cells in the blood was determined. The levels of erythrocytes, thrombocytes, leukocytes, neutrophil granulocytes, immature granulocytes, eosinophil granulocytes, basophil granulocytes, monocytes, and lymphocytes were measured and analyzed between non-survivors and survivors, as well as before and after SIRT. The effects of SIRT on the total blood cell count are presented in Table 4. The results show that the levels of leukocytes, neutrophil granulocytes, and immature granulocytes were significantly higher in non-survivors compared to survivors pre-T, while percentages of basophil granulocytes and monocytes were significantly lower in non-survivors versus survivors pre-T (*p* < 0.05, Table 4). While there were various significant differences between pre-T survivors versus post-T survivors, the only significant decrease observed between pre-T and post-T in non-survivors was found for the thrombocytes (*p* < 0.05, Table 4).

### 2.3. Plasma Levels of Circulating Cytokines in Relation to Selective Internal Radio Therapy and 60-Day Survival in HCC Patients

**Figure 2:**
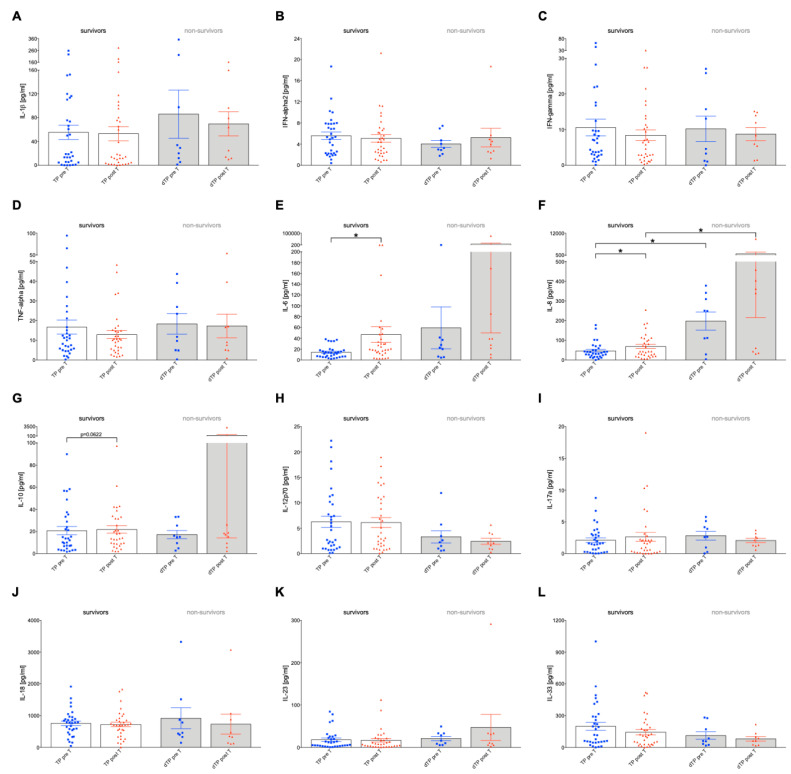
Analysis of plasma levels of circulating immunologic factors in relation to selective internal radio therapy (SIRT) and 60-day survival in HCC patients. The levels of various cytokines (**A**) Interleukin-1 beta, (**B**) Interferon-alpha2, (**C**) Interferon-gamma, (**D**) Tumor necrosis factor-alpha, (**E**) Interleukin-6, (**F**) Interleukin-8, (**G**) Interleukin-10, (**H**) Interleukin-12p70, (**I**) Interleukin-17a, (**J**) Interleukin-18, (**K**) Interleukin-23, and (**L**) Interleukin-33 in the plasma of HCC patients were analyzed using a bead-based LEGENDplex assay before therapy (pre-T) and after SIRT (post-T) in 60-day survivors and non-survivors. Data are expressed as the mean ± standard error of the mean. * *p* < 0.05 vs. indicated group.

Several cytokines have been measured to investigate their association with the treatment response to SIRT and/or 60-day survival rates, and to evidence changed inflammation or immune dysregulation in the HCC cohort (Figure 2). The focus has been on examining key immunologic factors such as TNF-alpha and interleukins (IL-6, IL-8, IL-10, IL12p70, IL-17a, IL-18, IL-23, IL-33, IL-1beta) and interferons (IFN-alpha2, IFN-gamma) (Figure 2) [21,22,23,24,25]. We report mainly statistically significant differences here; other changes are given in Figure 2. Analysis of IL-1beta, IFN-alpha, and IFN-gamma mediators of the inflammatory response showed no significant changes in the surviving and non-surviving groups before and after therapy (Figure 2A–C, *p* < 0.05). TNF-alpha, as an inflammation mediator, is produced and released by activated immune cells [22,26], and showed minimal changes in circulating levels both pre-T and post-T and between survivors and non-survivors (Figure 2D). IL-6 levels in the survivor group showed a significant increase pre-T versus post-T (Figure 2E, *p* < 0.05). Furthermore, a non-significant elevated post-T level of IL-6 was observed in the non-survivor group compared to pre-T levels and the survivor group (Figure 2E). IL-8 plays a role in the invasion, metastasis and prognosis of HCC [27,28]. A significant increase in IL-8 levels was observed in the survivor group post-T versus pre-T, while a general increase was detected in the non-survivor groups both pre-T and post-T group compared to the corresponding survivors group (Figure 2F, *p* < 0.05). Circulating IL-10 levels showed a non-significant increase in pre-T versus post-T survivors (Figure 2G, *p* < 0.0622). Between non-survivors and survivors as well as pre-T versus post-T, levels of other cytokines, which play a critical role in the immune response and regulation of inflammation [29] (such as IL-18, IL-23, IL12p70, and IL17a), showed no changes between survivors, non-survivors, and pre-T versus post-T cohorts (Figure 2H–L).

### 2.4. Verification of Isolated EVs

**Figure 3:**
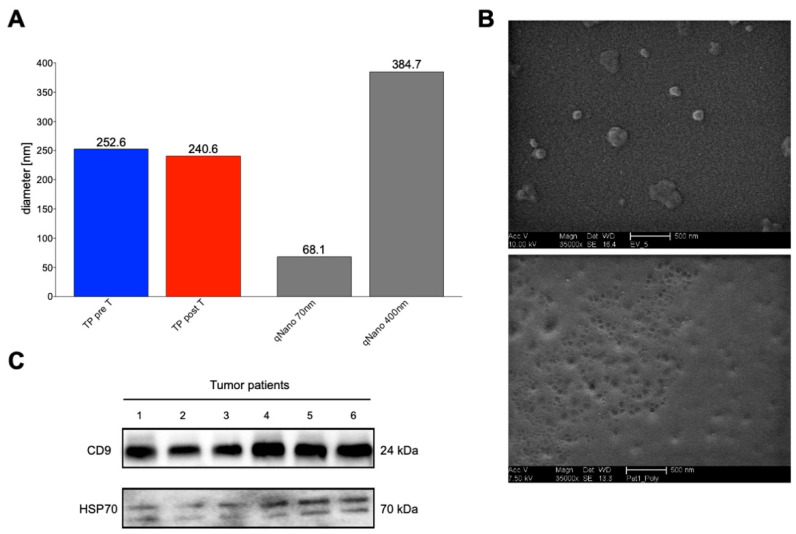
Assessment of the quality of isolated extracellular vesicles (EVs) from HCC patients. (**A**) Representative DLS-size distribution profile of calibration nanoparticles and isolated EVs collected in patients. (**B**) Scanning electron microscopy image of isolated EVs. The scale bar represents 500 nm (10,000×; AccV. 10 kV) and 500 nm (35,000×; AccV. 12 kV), respectively. (**C**) Representative Western blot assessing the protein content of isolated EVs from IZON fractions of HCC patients using the EVs markers CD9 and HSP70.

To evaluate if plasma fluid from the HCC cohort contains EVs and if IZON size-exclusion chromatography (SEC) is a suitable method for EV isolation, EVs from HCC patients were isolated using qEV SEC isolation, resulting in 24 fractions. The average diameter of isolated EVs determined by DLS was in the range of 100–400 nm, which is consistent with small and large EVs, mostly enriched in fractions 8–13 (Figure 3A). The average diameter of EVs from plasma samples was found to be 250 nm. Scanning electron microscopy was also used to confirm the size and morphology of EVs (Figure 3B). The sphere-shaped vesicles, which match the known morphology of large and small EVs, were validated by the data. After IZON column isolation, EVs were detected using Western blot, confirming the CD9 and HSP70 expression (Figure 3C).

### 2.5. SIRT-Driven Surface Immunologic Biomarker Expression on Plasma EVs

EVs, as a heterogeneous family of membrane-restricted vesicles, derive from the endosome or plasma membrane of their primary cells [30]. To evaluate the pre-T and post-T concentration of surface proteins on the EVs of HCC patients, a MACSPlex multiplex assay was performed. The diverse cell activation markers (e.g., leukocyte activation CD29, CD40, CD44, platelet activation marker, CD62P (P-selectin)), T and B cell adhesion protein markers (e.g., CD2, CD209, CD11c), cancer cell markers and progenitor markers (e.g., CD24, CD133, CD44, and CD142), and proteins that can indicate the cellular origin (e.g., CD19, CD4, CD56, CD14, CD3, CD42a, CD45 and CD31) were assessed.

**Figure 4:**
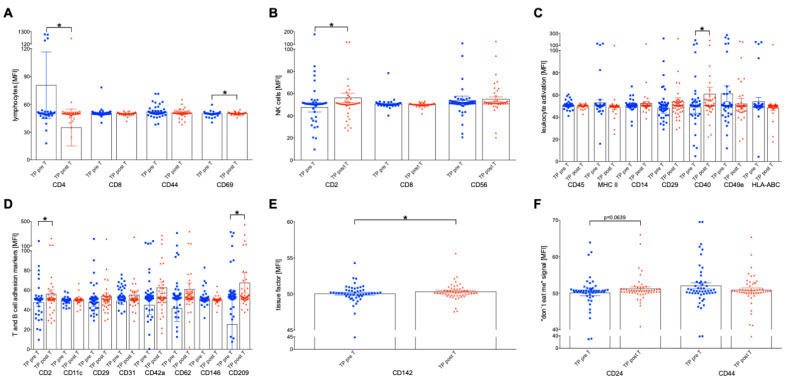
Procedure cluster of differentiation (CD) surface marker profile of immunologic extracellular vesicles (EVs) isolated from plasma from HCC tumor patients (TP) before (pre-T) and after selective internal radio therapy (post-T). EVs markers of (**A**) lymphocytes, (**B**) natural killer (NK) cells, (**C**) leukocyte activation, (**D**) T and B cell adhesion markers, (**E**) tissue factors, and (**F**) “don’t eat me signals” were investigated using a multiplex bead-based flow cytometry assay. The mean fluorescent intensity (MFI) is given as mean ± standard error of the mean. * *p* < 0.05 vs. indicated group.

The expression and comparison of lymphocytes, B cell, NK cell and platelet surface biomarkers on EVs were assessed pre-T and post-T for the entire HCC cohort. Interestingly, the signal of CD4+ positive EVs exhibited a significant decrease post-T compared to the pre-T levels (Figure 4A, *p* < 0.05). Conversely, the signal of CD69+ EVs showed a significant increase (Figure 4A, *p* < 0.05). Regarding B cell CD19+ and CD20+ associated EVs, no significant differences were observed between the pre-T and post-T. Similarly, no significant changes in the expression of NK cell-associated CD86+ EVs were observed between pre-T and post-T (Figure 4B). However, CD2+ EVs, differing from CD8+ and CD56+ EVs typically expressed on the surface of NK and T cells [31,32] demonstrated significantly elevated levels on the EVs’ surface following the therapy (Figure 4B, *p* < 0.05). There was a trend toward enhanced expression of platelet markers on EVs; however, this was not significant.

Additionally, the activation signals of the cells that were present on the EVs were examined and compared pre-T and post-T, complementing the assessment of cellular surface phenotype markers. CD40, which controls the immune response via various signaling cascades, was significantly enhanced on EVs post-T (Figure 4C, *p* < 0.05). B cell activation markers on EVs did not show significant differences, and no differences in the endothelial cell-originating EVs were detected. A significant increase in signal intensity was observed pre-T versus post-T on EVs expressing CD209, an adhesion molecule found on both T and B cells (Figure 4D, *p* < 0.05).

In addition to immunological EVs, the multiplex bead-based flow cytometry assay EV examined surface progenitor and stem cell markers, and cancer-associated biomarkers. CD142 exhibited a significant increase post-T versus pre-T (Figure 4E, *p* < 0.05). Furthermore, a comparison between pre-T and post-T revealed a non-significant increase in CD133 and CD24 expression (Figure 4F).

### 2.6. Surface Expression Markers on EVs in 60-Day Survivors and Non-Survivors

**Figure 5:**
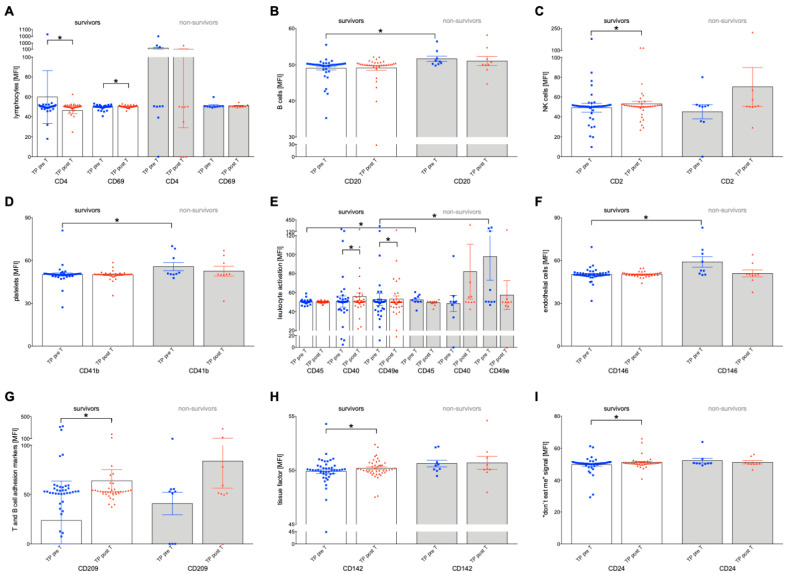
Cluster of differentiation (CD) surface marker profile of immunologic extracellular vesicles (EVs) isolated from plasma from HCC tumor patients (TP) before (pre-T) and after selective internal radiotherapy (post-T) in 60-day survivors and non-survivors. EVs markers for (**A**) lymphocytes, (**B**) B cells, (**C**) natural killer (NK) cells, (**D**) platelets, (**E**) leukocyte activation, (**F**) endothelial cells, (**G**) T and B cell adhesion markers (**H**), tissue factors, and (**I**) “don’t eat me signals” were investigated using a multiplex bead-based flow cytometry assay. The mean fluorescent intensity (MFI) is given as mean ± standard error of the mean. * *p* < 0.05 vs. indicated group.

In the group of surviving patients, significant differences were observed in the expression of EVs pre-T versus pos T. Only significant results are shown in Figure 5; otherwise, the differences were not significant. Only in the group of survivors compared to non-survivors were significant changes pre-T versus post-T observed (Figure 5). The expression of CD4 and CD69 on EVs significantly decreased post-T versus pre-T in survivors (Figure 5A, *p* < 0.05), while there was an increase in the expression of the markers CD2, CD40, CD49e, CD209, CD142 and CD24 (Figure 5C,E,G–I, *p* < 0.05). Furthermore, significant differences were found between survivors and non-survivors in the expression of the B cell-associated factor CD20 on EVs, platelet-associated factor CD41b on EVs, leukocyte-associated factor CD45, and endothelium-associated CD49e on EVs, as well as CD146 on EVs (Figure 5B,D–F, *p* < 0.05).

### 2.7. Correlation Analyses of Immune and Clinical Parameters with Each Other and with Outcome

A Spearman correlation analysis was performed to investigate the relationship between cell types, circulating cytokines, and EVs subtypes. The number of leukocytes before therapy positively correlated with the expression of CD49e EVs pre-T and with the concentration of IL-6 post-T (*p* < 0.05, Table 5). Leukocyte numbers and IL-8 level as well as CD41b, CD45, and CD146 expression on EVs did not correlate with each other. The thrombocytes counts correlated significantly and positively with the expression of cell adhesion molecule CD49e and platelet-associated CD41b on EVs pre-T (*p* < 0.05, Table 5). Plasma IL-8 and IL-6 levels positively correlated pre-T with each other (*p* < 0.05, Table 5). Leukocyte-associated CD45 correlated positively with CD49e and CD41b-positive EVs pre-T (*p* < 0.05, Table 5). In addition, pre-T and post-T CD45 EV expression correlated positively with endothelial cell-associated CD146 EVs expression (*p* < 0.05, Table 5). Pre-T CD49e EVs expression correlated positively with CD45 EVs and CD41b expression (*p* < 0.05, Table 5). In comparison to CD146 EVs, CD49e EVs showed a positive and significant correlation with CD49e (*p* < 0.05, Table 5). CD41b EVs had a significantly positive correlation with CD45, CD49e and CD146 EVS pre-T (*p* < 0.05, Table 5). After therapy, they were only significantly correlated with CD45, CD49e and CD146 EVs post-T (*p* < 0.05, Table 5). CD146 positive EVs positively correlated with CD45 EVs, CD49e and CD41b EVs pre-T and with CD146 EVs post-T (*p* < 0.05, Table 5). CD146 EVs post-T significantly correlated positively with CD45 and CD49 EVs post-T (*p* < 0.05, Table 5). Expression of B cell-associated CD20 EVs correlated significantly and positively with pre-T levels of IL-6 and IL-8 (*p* < 0.05, Table 5). Similarly, CD20 correlated significantly with pre-T CD45 and CD49, CD41b and CD146 Evs (*p* < 0.05, Table 5). Post-therapy CD20 expressing Evs positively correlated with post-T CD45 and CD41b and CD146 Evs (*p* < 0.05, Table 5).

To explore the results from the EV surface protein characterization, correlation analyses were performed with tumor size, received activity dose, tumor size and tumor fraction, interval until death, and 60-day survival pre-T and post-T (Table 6). Before treatment, there was a significant positive correlation between leukocyte numbers and tumor size (*p* < 0.05, Table 6). Similarly, a significant positive correlation was found between leukocyte concentration and tumor fraction and administered activity dose (MBq), as well as a significant correlation with survival (*p* < 0.05, Table 6). In the post-T phase, only a significant positive correlation between leukocyte numbers, administered radiation dose and negative correlation with the 60-day survival was detected (*p* < 0.05, Table 6). In addition to leukocyte concentration, thrombocyte count was also compared with tumor parameters. Significant correlations were found between tumor size, tumor fraction, and pre-T administered activity dose (MBq) (*p* < 0.05, Table 6). A significant correlation was also found between tumor size, administered activity dose (MBq), and tumor fraction post-T (*p* < 0.05, Table 6). For IL-6, no significant correlation was found between tumor size, tumor fraction, administered activity dose (MBq), interval to death (days), and 60-day survival. IL-8 correlated significantly pre-T with the tumor fraction, and furthermore, a significant negative correlation with 60-day survival was found (*p* < 0.05, Table 6). After the therapy, the plasma concentration of IL-8 correlated significantly negatively with the interval to death (days) and 60-day survival (*p* < 0.05, Table 6). In the group of analyzed EVs, CD45 and CD49e expression negatively correlated with the 60-day survival (*p* < 0.05, Table 6). No correlation with patient parameters was observed after therapy. Similarly, platelet-associated CD41b EVs showed a negative correlation with 60-day survival pre-T (*p* < 0.05, Table 6). The endothelial marker CD146 also negatively correlated with patient survival (*p* < 0.05, Table 6). Additionally, CD20-bearing EVs negatively correlated with patient survival (*p* < 0.05, Table 6).

## 3. Discussion

Hepatocellular carcinoma has become increasingly significant in Western countries due to its rising incidence [2,5]. The majority of HCC cases are diagnosed at a non-respectable stage [5], highlighting the need for minimally invasive methods and personalized oncological decision making. In recent years, multimodal treatment approaches such as TACE, high-dose brachytherapy, and microwave ablation (MWA) have been developed for HCC treatment [3,33,34]. Additionally, it is crucial to identify patients who are suitable candidates for SIRT to minimize side effects and optimize treatment response. However, the current selection criteria for SIRT primarily rely on imaging, dosimetry, and liver function, with limited consideration of individual tumor biology and systemic immunology [35]. Here, the role of EVs in plasma or serum before and after tumor therapy, including SIRT, can be easily obtained in sufficient quantities. This makes them attractive candidates as potential biomarkers, supplementing cell counts and cytokines levels in the serum, to provide additional insights into the patient’s response to treatment.

In our study, we aimed to gain new insights into the characteristics and origin of Evs in HCC patients before and after SIRT. We assessed 37 EV markers, cell counts and inflammatory cytokines in the treated patients. Our objectives were to understand the relationship between these markers, their response to therapy, and their potential association with survival outcomes. We observed significant changes in EV protein expression, cytokine levels, and cell counts following therapy. These alterations appeared to be influenced by the proinflammatory response triggered by the therapy, as well as the tumor burden and liver function (Table 2 and Table 3). Specifically, we observed an increase in the total number of granulocytes and neutrophils, accompanied by a decrease in lymphocytes and platelets. These differences were more pronounced when comparing patients with higher and lower survival rates (Table 2 and Table 4).

Interestingly, proinflammatory and immunomodulatory cytokines, such as IL-6 and IL-8, significantly increased as a result of the therapy. Furthermore, IL-8 exhibited a negative correlation with patient survival. These cytokines have been implicated as pro-malignant mediators in various tumor types, and have been associated with shorter overall survival in previous studies [36,37,38,39]. In HCC, IL-8 has been shown to promote different stages of tumor progression, including the invasion of cancer cells into blood vessels, by upregulating the PI3K/Akt pathway [27,40]. The increase in IL-6 and IL-8 during therapy could potentially be explained by the fact that SIRT enhances the IL-6-mediated acute phase response in the liver [37]. Additionally, IL-8 has been linked to liver dysfunction, and an increase in IL-8 levels could indicate heightened accumulation of macrophage in the liver due to SIRT [36,41]. These findings shed light on the potential mechanisms underlying the effects of SIRT on cytokine regulation, and their impact on HCC progression and patient outcomes.

In addition to the increased IL-6 and IL-8 levels, our study also revealed an elevation in various immune cell-derived markers, including CD2, CD40, CD69, and CD209. These findings indicate a proinflammatory status induced by SIRT. Immune cell-derived EVs play crucial roles in intercellular communication and regulation of specific mechanisms of both adaptive and innate immune responses [42,43,44]. Similar to our results, previous research by Tung et al. demonstrated the influence of EVs on dendritic cell activity and mobility through the study of dendritic cell interaction with exosomes derived from regulatory T cells (Tregs). Another interesting finding is the role of CD40 as a costimulatory molecule [45]. Verma et al. discovered that hepatocytes treated with ethanol released caspase-dependent EVs enriched in CD40 ligand (CD40L), which induced proinflammatory responses in macrophages. Mice lacking CD40 were protected from alcohol-induced liver inflammation [46]. In a similar experimental setup, elevated levels of proinflammatory EVs were observed in the circulation of patients with cholangiocarcinoma (CCA) undergoing SIRT, which aligns with our findings [47]. Future studies should investigate whether there are variations between different tumor entities. The liver, like other barrier sites, is enriched with natural killer (NK) cells and innate T cells [48,49]. The SIRT-induced expression of CD69 and CD209 suggests the release of tissue-resident immune cells. Conversely, we observed a decrease in the expression of CD4+ EVs, accompanied by a decrease in lymphocytes. The biological effects and functions of CD4+ T cell-derived EVs in cancer are still not fully understood. One possibility is that CD4+ T cell EVs enhance B cell responses, with CD40L being involved in the mediation of this response [50].

In our study, we made an intriguing discovery regarding the predictive correlation of certain EV markers with patient survival. as shown in Table 6. Notably, CD20, CD45, CD49e, and CD146 expressing EVs exhibited this predictive correlation. Of particular interest is CD146, which has been independently described as a promalignant mediator and a predictor of poor prognosis in HCC [51]. In our study, we demonstrated that CD146 is also detectable at the EV level, and higher levels of CD146+ EVs were associated with worse patient outcomes. CD20+ EV presence also showed significant findings. Moreover, the expression of CD20+ on EVs correlated with IL-6 and IL-8 levels. This suggests that CD20+ EVs could serve as a potent marker, similar to how CD20+ B cells have been found to correlate with overall and recurrence-free survival in HCC [52]. Elevated levels of CD20+ EVs, along with increased IL-6, IL-8, and B cell levels, may be associated with an increased incidence of organ failure [53].

In our study, we observed the presence of the hematopoietic marker CD45 and the platelet marker CD41b on EVs, and these markers showed strong correlations with other EV markers such as CD20, CD49e, and CD146. This suggests that a significant proportion of EVs may be released by immune cells or platelets (Table 5). The expression of CD49e, a membrane-bound alpha-5 integrin, further supports the notion that most EVs are formed through direct outward budding or pinching of the cell’s plasma membrane, which is a common mechanism of EVs formation [54,55,56]. In our study, we observed that SIRT not only had an impact on the immune system, but also on platelet count and the expression of platelet-associated proteins on EVs. Liver dysfunction resulting from HCC strongly affects the coagulation system [57,58,59]. Changes in platelets and alterations in EV expression of platelet-associated proteins can be attributed to the compromised liver function observed in advanced HCC patients [60]. Among other factors contributing to thrombocytosis, the increased expression of tissue factor CD142 and CD41bmay also be involved [47,61,62,63]. Understanding the interplay between SIRT, platelet function, and EV expression may provide insights into the complex relationship between cancer, liver function, and coagulation in HCC patients, and should be studied further in future studies. Our study focused on specific markers and their response to therapy, lacking comprehensive data necessary for individual treatment evaluation. While providing valuable insights into marker changes pre- and post-therapy, we acknowledge the need for more extensive analysis to definitively assess treatment response. Elevated liver function (AST, GGT), leukocytes, and tumor volume imaging correlated with reduced patient survival. Our research aimed to retrospectively evaluate EVs’ therapeutic prognostic efficacy. It is essential to approach these findings critically, and we acknowledge that a single statistically significant correlation is not sufficient to establish a causal relationship. We are still in the early stages of understanding the role of EVs in effective patient prognosis. However, our study has identified several EV markers that warrant further investigation. The results highlight the significant prognostic potential of EVs as additional parameters for making oncological decisions, in addition to imaging, clinical parameters, and conventional laboratory measurements such as liver function tests. We anticipate that future research will continue to explore the potential of EVs as part of liquid profiling in cancer diagnosis and prognosis. However, it is important to approach findings with caution, since further studies involving larger cohorts of patients and different therapeutic approaches are necessary to fully evaluate the prognostic potential of EVs. Only through rigorous and extensive investigation can we confidentially determine the clinical relevance and utility of EVs as prognostic markers in cancer patients.

## 4. Materials and Methods

### 4.1. Ethics

This study was conducted at the University Hospital of the University of Magdeburg with the approval of the Institutional Ethics Committee (SWARM RAD298) and in accordance with the Declaration of Helsinki. All enrolled patients gave written informed consent.

### 4.2. Study Settings and Population

Some 50 (female n = 10, male n = 40, median age 69 (62–79) years) patients underwent SIRT due to suffering with HCC. The inclusion criteria were as follows: (i) patients with HCC, (ii) indication for radioembolization (RE)/SIRT, (iii) chemotherapy and cortisone therapy paused for a minimum of two weeks before admission, and (iv) >18 years of age. The exclusion criteria were (i) life expectancy < 3 months; (ii) hepatic tumor load > 70%; (iii) chronic infections, patients with infectious diseases such as HIV and hepatitis B and C; (iv) pronounced ascites; (v) contraindications for angiography, MRI contrast medium (Gd-EOB-DTPA), X-ray contrast medium, MRI and CT; (vi) severe cardiovascular diseases (NYHA III/IV); (vii) thrombotic or embolic events in the last 6 months (stroke/TIA); (viii) immunosuppression or HIV, especially cortisone long-term therapy; and (ix) autoimmune diseases or chronic inflammatory bowel diseases.

### 4.3. Technique of 90Y-Radioembolization

A detailed description of RE/SIRT is given elsewhere [64]. RE was performed using Yttrium-90 (90Y) resin microspheres (SIR-Spheres^®^, Sirtex Medical, Lane Cove, Australia). Before RE, the hepatic arterial tree and the arterial feeders to the gastrointestinal tract were identified using angiography. To isolate the hepatic arterial blood supply, the gastroduodenal and right gastric arteries, as with any other gastrointestinal tract feeder, were embolized using coils. Subsequently, 99mTc-MAA (150 MBq, 99mTc-LyoMAA, COVIDien, Neustadt/Donau, Germany) was injected into the hepatic artery, and the distribution and the extent of hepatopulmonary shunting were visualized using a gamma camera (E.CAM 180, Siemens, Erlangen, Germany). To detect extrahepatic non-target seeding of 99mTc-MAA, a single-photon emission computed tomography (SPECT) scan of the upper abdomen was performed. The body surface area (BSA) method was used to calculate the activity of the 90Y resin microspheres [65]. During the two-week period following the previous procedure, a temporary transfemoral catheter was inserted into the proper hepatic artery, and 90Y resin microspheres were then selectively delivered into the hepatic arteries though the catheter (Figure 1A–F).

### 4.4. Data Acquisition and Blood Sampling

Laboratory evaluations were performed one day prior (24 h) and two days after (48 h) SIRT. Blood samples were collected in ethylenediaminetetraacetic acid-rinsed (EDTA) tubes (Becton Vacutainer, Becton Dickinson Diagnostics, Aalst, Belgium) before (pre-T) and after (post-T) SIRT. The EDTA blood was kept at room temperature (RT), avoiding agitation. If the SIRT procedure was repeated in patients, plasma was also drawn before and after the following therapies. Blood was centrifuged at 2000× *g* for 15 min at 4 °C, and plasma was stored at −80 °C until the isolation of EVs and sample analyses (Figure 1G). Blood counts (leukocytes, erythrocytes, red cell distribution width, thrombocytes, neutrophil granulocytes, immature granulocytes, eosinophil granulocytes, basophil granulocytes, lymphocytes, and monocytes) were determined in the clinical routine, as well as the coagulation parameters (quick value, international normalized ratio (INR), partial thromboplastin time (PTT) and thrombin time), clinical chemistry (creatinine, urea, uric acid, bilirubin, albumin, alanine aminotransferase, aspartate aminotransferase, alkaline phosphatase, and gamma-glutamyl transferase), and C-reactive protein (CRP). Serum tubes (clinical chemistry and CRP), EDTA tubes (blood count), and citrate tubes (coagulation) (Becton Vacutainer, Becton Dickinson Diagnostics, Aalst, Belgium) were used for the clinical blood collection.

### 4.5. Isolation of Extracellular Vesicles by Size Exclusion Chromatography

EVs were isolated from plasma using IZON original qEV 70 nm columns (Gen 1., Izon, Christchurch, New Zealand) and were vertically positioned at room temperature. The columns were equilibrated with 20 mL particle-free degassed and 0.22-µm-filtered PBS. In parallel, the plasma samples stored at −80 °C were thawed in a water bath (~20 °C), and then centrifuged for 60 min at 15,000× *g* at 4 °C for the separation of large microparticles according to EV isolation guidelines. Subsequently, the plasma samples were diluted to 500 µL with 500 µL particle-free PBS, and the sample was overlaid on the qEV size exclusion column followed by elution with particle-free PBS. As soon as the sample volume was taken up by the column, 10 mL filtered PBS was added to the top of the column tube. The following fractions were collected: F0 (1 mL = void volume of the column) and F1 to F8 (500 µL each), according to the manufacturer’s instructions.

### 4.6. EV Measurement via FACS

The isolated EVs were incubated with MACSPlex capture beads from a MACSPlex Exosome kit (#130-108-813, Miltenyi Biotec, Bergisch Gladbach, Germany), overnight, on an orbital shaker (450 rpm, RT). On the next day, 1 mL of MACSPlex buffer was added (MACSPlex Exosome kit) and the sample was centrifuged (3000× *g*, 5 min, RT). Then, 1 mL of the supernatant was discarded, and 135 µL was left in the tube; 15 µL of MACSPlex detection reagent “master mix” was added to the leftover. The “master mix” was made using a combination of 5 µL detection reagent for CD9, 5 µL detection reagent for CD63, and 5 µL detection reagent for CD81 (MACSPlex Exosome kit). The sample was incubated for one hour at RT, and afterwards centrifuged (3000× *g*, RT, 5 min). After centrifugation, 1 mL of supernatant was discarded, and 1 mL of MACSPlex buffer was added. Then, the sample was incubated in the dark on an orbital shaker (450 rpm) for 15 min. The incubated sample was centrifuged (3000× *g*, RT, 5 min). Then, 1 mL of the supernatant was discarded. The leftover was resuspended, transferred into a fluorescence-activated cell sorting (FACS) tube, and analyzed via flow cytometry. Data assessed by flow cytometry were normalized to internal markers provided in the assay, as suggested by the manufacturer, and a blank control (beads and Macsplex buffer) was used to deduct the background signal. Then, the APC median signal intensities between the groups were compared.

### 4.7. Chemokine Assay

Cytokines were measured in patients’ plasma pre-T and post-T using LEGENDplex™ bead-based immunoassays (BioLegend, San Diego, CA, USA). IL-1 beta, IL-1β, IFN-alpha2, IFN-α2, IFN-gamma, IFN-γ, TNF-alpha, MCP-1, IL-6, IL-8, IL-10, IL-12p70, IL-17A, IL-18, IL-23 and IL-33 α were determined using the Human Inflammation Panel 1 cytokine panel (13-plex, Biolegend) according to the manufacturer’s instructions. Samples were assessed using BD FACSCelesta™, and data was analyzed using BioLegend’s LEGENDplex™ data analysis software version 8 (Figure 2).

### 4.8. Western Blotting

The total protein concentration obtained from EVs was quantified using Lowry Protein Assay [66]. Then, 10 µg of the isolated EV protein samples were lysed using sodium dodecyl sulfate (SDS) sample buffer (200 mM Tris-HCL (pH 6.8); 10% SDS; 0.4% bromophenol blue; 40% glycerol), and subsequently separated via SDS (12%) gel electrophoresis (SDS-PAGE). After the proteins were transferred on 0.45 µm PVDF membranes (Cytiva, 10600023, Marlborough, MA, USA), the membranes were blocked (5% milk powder and 0.1% tween in tris-buffered saline (TBS)) for one hour at RT. The following primary antibody incubation was performed overnight at 4 °C. To assess the presence of EVs, antibodies against CD9 (#312102 Biolegend, San Diego, CA, USA) and HSP70 (#sc-32239, Santa Cruz, Dallas, TX, USA) were applied, e.g., goat-anti-mouse-horseradish peroxidase (HRP) [47]. For detection of the protein signal, anti-mouse-horseradish peroxidase (HRP)-coupled secondary antibodies were incubated for one hour at RT (1:1000 dilution, polyclonal, ab205719, Abcam, Cambridge, UK). Subsequently, enhanced chemiluminescence (Millipore WBKLS0500, Merck KGaA, Burlington, MA, USA) detection and visualization were implemented and detected with an Octoplus QPLEX imager from NH DyeAgnostics (Halle, Germany). As in previous works, a semi-quantitative analysis of Western blot signals was assessed using ImageJ software Version 1.53t [47].

### 4.9. Dynamic Light Scattering

The extracted EVs were diluted to 1:100 using filtered and degassed Dulbecco’s phosphate-buffered saline (DPBS). The extra vesicle-containing cuvette (67.745, Sarstedt AG & Co. KG, Nümbrecht, Germany) was measured after laser and temperature equilibration of the DLS device (Zetasizer Nao, ZEN 3600 Malvern Instruments LTD., Malvern, UK) [47]. For reproducibility and standardization, the parameter values were fixed as follows: a laser wavelength (nm) of 632.8, a material refraction index of blood EVs of 1.39, and a dispersant refraction index of water of 1.330. The size or homogeneity of EVs was recognized through the radius or the % intensity, respectively [47,67]. Measurement with DLS was conducted with ten acquisitions of 5 s. N represents the number of patients, and n represents the data collected from each sample (n = 9, pre-T: N = 6, n = 9, post-T: N = 6, n = 9).

### 4.10. Scanning Electron Microscopy (SEM)

To obtain an overview of the composition, size, and morphology of EV samples, isolated EVs were prepared for scanning electron microscopy (SEM) analysis. For preparation, carbon-conductive PELCO Tabs™ (Ted Pella, Inc.) were cleaned with ethanol and ultrapure water, and then coated with 100 µg/mL poly-D-lysine (Millipore A003E, Merck KgaA, Darmstadt, Germany) solution for 4 h at 37 °C [23]. Then, the EV samples were loaded onto poly-D-lysine-coated carbon-conductive tabs, and incubated overnight at RT. Subsequently, the EVs were fixed with 4% formaldehyde and incubated for 15 min at RT. After washing with ultrapure water, a gradual dehydration from 70% to 100% ethanol in water with a 10% concentration increment step every 5 min followed [68,69]. The EV samples were then coated with gold (<10 nm) to increase the image contrast, enhance the electric conductivity, and avoid surface charging. The images of samples were captured with a scanning electron microscope (an FEI Scios DualBeam equipped with an EDAX EDS system, Thermo Fischer Scientific Inc., Waltham, MA, USA) at 10–12 KeV voltages and 10,000× and 35,000× resolution.

### 4.11. Statistics

GraphPad Prism 6.0 software (GraphPad Software Inc. San Diego, CA, USA) was used for statistical analysis. The normality of all data was verified using a Kolmogorov–Smirnov test. Data are given as mean ± standard error of the mean, unless otherwise indicated. The differences between the two groups were determined using unpaired non-parametric Mann–Whitney rank tests. The statistically significant differences between data pre-T versus post-T were assessed using paired non-parametric Wilcoxon matched-pairs signed rank tests. Sex distribution among the groups was compared using a Chi-squared test. Correlation analyses were performed by determining the Spearman correlation significance and Spearman r. A *p* value below 0.05 was considered statistically significant [47].

## Figures and Tables

**Table 1:** Descriptive baseline characteristics and clinical parameters of the study population obtained before selective internal radiotherapy. Median values with the 75% percentile are given. Abbreviations: INR, international normalized ratio; PTT, partial thromboplastin time.

Variables	n = 50
Age [median (range)]	69.00 (62.75–79.00)
Sex (female, n)	10
Total liver volume (cm^3^) [median (range)]	1955 (1619–2565)
Tumor volume (cm^3^) [median (range)]	175.50 (84.50–563.00)
Tumor fraction (%) [median (range)]	9.10 (4.43–26.80)
Administered activity dose (MBq) [median (range)]	1180.00 (976.50–1475.00)
Creatinine (umol/L) [median (range)]	81.50 (64.75–95.25)
Urea (mmol/L) [median (range)]	4.85 (3.58–7.50)
Uric acid (umol/L) [median (range)]	338.00 (266.50–429.00)
Bilirubin (umol/L) [median (range)]	10.20 (8.03–15.28)
Albumin (g/L) [median (range)]	39.55 (36.50–43.10)
Alanine aminotransferase (umol/s·L) [median (range)]	0.63 (0.41–1.00)
Aspartate aminotransferase (umol/s·L) [median (range)]	0.88 (0.64–1.35)
Alkaline phosphatase (umol/s·L) [median (range)]	2.26 (1.67–3.53)
Gamma-glutamyl transferase (umol/s·L) [median (range)]	3.41 (1.68–6.80)
Quick value (%) [median (range)]	86.00 (78.50–94.00)
INR [median (range)]	1.080 (1.030–1.135)
PTT (s) [median (range)]	29.30 (27.30–32.10)
Thrombin time (s) [median (range)]	16.90 (16.35–17.65)
C-reactive protein (mg/L) [median (range)]	12.43 (5.32–18.03)

**Table 2:** The blood counts before (pre-T) and after selective internal radiotherapy (post-T). Median values with the 75% percentile are given. A nonparametric Wilcoxon matched-pairs signed rank test was performed, and a *p* value below 0.05 was considered statistically significant.

Variables	Pre-T	Post-T	*p* Value
Leukocytes (Gpt/L) [median (range)]	6.67 (5.34–8.96)	7.13 (5.08–9.75)	0.0934
Erythrocytes (Tpt/L) [median (range)]	4.27 (3.64–4.64)	4.28 (3.74–4.57)	**0.0010**
Thrombocytes (Gpt/L) [median (range)]	177.50 (118.30–246.80)	146.00 (98.00–210.50)	**<0.0001**
Neutrophil granulocytes (%) [median (range)]	68.70 (62.00–75.00)	74.50 (67.05–82.18)	**0.0027**
Neutrophil granulocytes (Gpt/L) [median (range)]	5.25 (3.48–7.60)	6.62 (3.79–9.68)	**0.0046**
Immature granulocytes (%) [median (range)]	0.00 (0.00–0.01)	0.00 (0.00–0.40)	0.5000
Immature granulocytes (Gpt/L) [median (range)]	0.05 (0.02–0.10)	0.04 (0.02–0.06)	0.6240
Eosinophil granulocytes (%) [median (range)]	2.00 (1.00–3.00)	1.00 (0.00–1.96)	0.1289
Eosinophil granulocytes (Gpt/L) [median (range)]	0.13 (0.09–0.21)	0.04 (0.03–0.05)	0.1060
Basophil granulocytes (%) [median (range)]	1.00 (0.50–1.00)	0.25 (0.00–1.00)	**0.0156**
Basophil granulocytes (Gpt/L) [median (range)]	0.06 (0.04–0.06)	0.04 (0.03–0.05)	0.1113
Lymphocytes (%) [median (range)]	20.00 (15.00–23.80)	13.15 (9.60–19.05)	**0.0005**
Lymphocytes (Gpt/L) [median (range)]	1.43 (1.24–1.79)	1.07 (0.68–1.45)	**0.0027**
Monocytes (%) [median (range)]	9.00 (6.50–11.40)	9.40 (6.88–11.18)	0.2500
Monocytes (Gpt/L) [median (range)]	0.68 (0.54–0.97)	0.74 (0.51–1.09)	**0.0156**

**Table 3:** Descriptive baseline characteristics and clinical laboratory parameters of the study population obtained before (pre-T) and after selective internal radiotherapy (post-T) in the 60-day survivor group versus the non-survivor group. Median values with the 75% percentile are given. Abbreviations: INR, international normalized ratio; PTT, partial thromboplastin time. Significant changes are indicated as follows: (a) pre-T survivors versus pre-T non-survivors, (b) post-T survivors versus post-T non-survivors, (c) pre-T survivors versus post-T survivors, and (d) pre-T non-survivors versus post-T non-survivors. An unpaired non-parametric Mann–Whitney rank test was applied for a and b, and paired a non-parametric Wilcoxon matched-pairs signed rank test was performed for c and d. Sex distribution among the groups was compared using a Chi-squared test. A *p* value less than 0.05 was considered statistically significant.

Variables	Pre-T		Post-T		
	Survivors (n = 41)	Non-Survivors (n = 9)	Survivors (n = 41)	Non-Survivors (n = 9)	*p* Value < 0.05
Age [median (range)]	69.00 (62.75–79.00)	70.00 (62.00–79.50)	69.00 (62.50–79.00)	70.00 (62.00–79.50)	
Sex (female, n)	9	1			
Total liver volume (cm^3^) [median (range)]	1775 (1445–2469)	2790 (1860–3332)			a
Tumor volume (cm^3^) [median (range)]	163.50 (65.20–394.00)	690.30 (133.10–1836.00)			
Tumor fraction (%) [median (range)]	5.40 (3.10–16.83)	27.40 (7.00–42.68)			
Administered activity dose (MBq) [median (range)]	1176.00 (968.30–1392.00)	1438.00 (1010.00–1579.00)			
Creatinine (umol/L) [median (range)]	83.00 (65.50–97.00)	72.00 (60.50–88.50)	82.00 (68.00–98.75)	87.00 (73.00–108.00)	d
Urea (mmol/L) [median (range)]	4.90 (3.45–7.50)	4.80 (4.10–7.20)	6.00 (4.56–8.73)	8.10 (5.25–9.90)	c
Uric acid (umol/L) [median (range)]	338.00 (271.80–406.50)	317.00 (249.00–451.00)	332.50 (258.30–387.80)	395.00 (251.50–530.00)	
Bilirubin (umol/L) [median (range)]	9.90 (7.85–15.35)	12.40 (8.00–15.85)	13.50 (8.68–18.95)	17.80 (9.90–23.55)	c, d
Albumin (g/L) [median (range)]	39.90 (36.75–43.20)	39.40 (34.00–42.15)	37.10 (33.68–40.83)	36.60 (29.70–39.30)	c, d
Alanine aminotransferase (umol/s·L) [median (range)]	0.62 (0.41–1.02)	0.79 (0.52–1.07)	0.63 (0.43–0.99)	0.72 (0.55–2.16)	
Aspartate aminotransferase (umol/s·L) [median (range)]	0.85 (0.64–1.26)	1.23 (0.65–2.11)	0.77 (0.59–1.45)	2.10 (1.06–8.10)	b, d
Alkaline phosphatase (umol/s·L) [median (range)]	2.15 (1.54–3.07)	3.64 (2.20–5.22)	1.89 (1.37–2.66)	3.10 (1.69–4.03)	a, c
Gamma-glutamyl transferase (umol/s·L) [median (range)]	2.90 (1.67–6.45)	4.84 (2.03–20.50)	2.84 (1.67–6.12)	3.51 (1.73–23.55)	c
Quick value (%) [median (range)]	85.00 (78.25–93.50)	86.00 (78.00–98.00)	81.00 (74.00–94.50)	75.00 (57.50–92.50)	
INR [median (range)]	1.085 (1.033–1.138)	1.080 (1.010–1.140)	1.120 (1.030–1.170)	1.170 (1.040–1.360)	
PTT (s) [median (range)]	29.25 (27.50–32.65)	30.50 (25.90–30.85)	28.70 (27.05–31.10)	30.00 (26.80–33.65)	
Thrombin time (s) [median (range)]	16.90 (16.35–17.65)	16.45 (15.65–17.98)	17.60 (16.85–18.45)	18.45 (16.75–19.38)	d
C-reactive protein (mg/L) [median (range)]	12.20 (3.58–14.70)	21.30 (8.80–58.80)	6.50 (3.72–12.98)	18.70 (8.70–69.25)	b

**Table 4:** The blood counts of the study population obtained before (pre-T) and after selective internal radiotherapy (post-T) in the 60-day survivor group versus the non-survivor group. Median values with the 75% percentile are given. Abbreviations: INR, international normalized ratio; PTT, partial thromboplastin time. Significant changes are indicated as follows: (a) pre-T survivors versus pre-T non-survivors, (b) post-T survivors versus post-T non-survivors, (c) pre-T survivors versus post-T survivors, and (d) pre-T non-survivors versus post-T non-survivors. An unpaired non-parametric Mann–Whitney rank test was applied for a and b, and a paired non-parametric Wilcoxon matched-pairs signed rank test was performed for c and d. Sex distribution among the groups was compared using a Chi-squared test. A *p* value less than 0.05 was considered statistically significant.

Variables	Pre-T		Post-T		
	Survivors (n = 41)	Non-Survivors (n = 9)	Survivors (n = 41)	Non-Survivors (n = 9)	*p* Value < 0.05
Leukocytes (Gpt/L) [median (range)]	6.22 (5.28–7.84)	11.20 (6.94–12.85)	6.66 (5.04–9.13)	10.90 (6.81–14.45)	a, b
Erythrocytes (Tpt/L) [median (range)]	4.26 (3.59–4.61)	4.43 (4.03–4.92)	4.25 (3.65–4.52)	4.48 (4.06–4.70)	c
Thrombocytes (Gpt/L) [median (range)]	171.00 (110.50–233.00)	244.00 (159.50–467.50)	145.00 (97.50–211.30)	184.00 (97.50–354.00)	c, d
Neutrophil granulocytes (%) [median (range)]	67.00 (61.90–71.00)	76.30 (73.85–81.45)	72.90 (65.88–77.33)	87.15 (82.18 –92.80)	a, b, c
Neutrophil granulocytes (Gpt/L) [median (range)]	4.88 (2.93–6.38)	9.85 (8.58–13.95)	5.83 (3.75–7.81)	12.83 (9.24–19.08)	a, b, c
Immature granulocytes (%) [median (range)]	0.00 (0.00–0.01)	0.01 (0.00–1.05)	0.00 (0.00–0.40)	0.01 (0.00–1.88)	
Immature granulocytes (Gpt/L) [median (range)]	0.04 (0.01–0.07)	0.16 (0.09–0.17)	0.04 (0.02–0.05)	0.21 (0.08–0.38)	a, b
Eosinophil granulocytes (%) [median (range)]	2.00 (1.00–3.00)	1.25 (0.30–3.25)	1.00 (0.08–2.25)	0.10 (0.00–0.28)	
Eosinophil granulocytes (Gpt/L) [median (range)]	0.12 (0.09–0.21)	0.16 (0.07–0.57)	0.08 (0.02–0.15)	0.03 (0.01–0.05)	
Basophil granulocytes (%) [median (range)]	1.00 (0.70–1.00)	0.40 (0.08–0.58)	0.55 (0.00–1.00)	0.15 (0.03–0.20)	a, c
Basophil granulocytes (Gpt/L) [median (range)]	0.05 (0.04–0.06)	0.06 (0.04–0.08)	0.05 (0.03–0.05)	0.03 (0.02–0.04)	
Lymphocytes (%) [median (range)]	21.00 (17.00–25.40)	15.30 (8.20–19.63)	14.65 (11.68–19.40)	4.65 (2.08–10.60)	b, c
Lymphocytes (Gpt/L) [median (range)]	1.42 (1.23–1.64)	1.86 (1.35–2.46)	1.13 (0.72–1.49)	0.68 (0.38–1.26)	c
Monocytes (%) [median (range)]	9.40 (8.00–12.00)	5.75 (4.63–7.25)	9.80 (8.30–11.33)	5.15 (2.90–9.88)	a
Monocytes (Gpt/L) [median (range)]	0.68 (0.54–0.77)	0.81 (0.54–1.14)	0.74 (0.51–0.93)	0.93 (0.44–1.23)	c

**Table 5:** The correlation analyses between the immune cell count, interleukin (IL)-6 and IL-8, and extracellular vesicles (EVs) were performed by determining the Spearman correlation significance and Spearman r. Expression levels of cluster of differentiation (CD) proteins on the surface of EVs isolated from hepatocellular carcinoma patients before (pre-T) and after selective internal radiotherapy (post-T). A *p* value below 0.05 was considered statistically significant.

Parameter		IL-6 (pg/mL)				IL-8 (pg/mL)				Leukocyte Activation CD45 EVs				Leukocyte Activation CD49e EVs				Platelets CD41b EVs				Endothelial Cells CD146 EVs			
	Time Point	Pre-T		Post-T		Pre-T		Post-T		Pre-T		Post-T		Pre-T		Post-T		Pre-T		Post-T		Pre-T		Post-T	
		r	*p* Value	r	*p* Value	r	*p* Value	r	*p* Value	r	*p* Value	r	*p* Value	r	*p* Value	r	*p* Value	r	*p* Value	r	*P* Value	r	*p* Value	r	*p* Value
**Leukocytes (Gpt/L)**	pre-T	0.182	0.255	0.101	0.525	0.223	0.162	−0.003	0.983	0.037	0.797	−0.016	0.912	0.281	**0.048**	0.155	0.282	0.186	0.196	0.087	0.547	0.179	0.213	0.102	0.481
	post-T	-	-	0.334	**0.033**	-	-	0.058	0.720	-	-	−0.130	0.373	-	-	0.261	0.070	-	-	0.120	0.411	-	-	0.081	0.582
**Thrombocytes (Gpt/L)**	pre-T	−0.055	0.732	0.049	0.756	0.180	0.260	0.105	0.508	−0.081	0.578	−0.011	0.937	0.061	**<0.001**	0.038	0.796	0.112	**<0.001**	−0.028	0.850	0.133	0.358	0.171	0.235
	post-T	-	-	0.044	0.783	-	-	0.114	0.480	-	-	−0.107	0.464	-	-	0.043	0.770	-	-	−0.090	0.538	-	-	0.021	0.887
**IL-6 (pg/mL)**	pre-T	-	-	0.046	0.777	0.420	**0.006**	0.133	0.406	0.164	0.306	−0.111	0.490	0.163	0.307	−0.134	0.402	0.199	0.211	0.139	0.385	0.075	0.643	−0.160	0.316
	post-T	-	-	-	-	-	-	0.051	0.748	-	-	0.108	0.497	-	-	0.239	0.127	-	-	0.243	0.121	-	-	0.130	0.412
**IL-8 (pg/mL)**	pre-T	0.420	**0.006**	−0.165	0.304	-	-	0.545	**<0.001**	0.027	0.869	−0.149	0.351	0.113	0.484	−0.128	0.427	0.294	0.062	0.097	0.545	0.134	0.403	−0.213	0.180
	post-T	-	-	0.051	0.748	-	-	-	-	-	-	−0.260	0.097	-	-	−0.188	0.233	-	-	0.098	0.538	-	-	−0.151	0.339
**Leukocyte activation CD45 EVs**	pre-T	0.164	0.306	−0.010	0.950	0.027	0.869	−0.049	0.759	-	-	0.497	**<0.001**	0.422	**0.001**	−0.039	0.787	0.442	**0.001**	0.143	0.322	0.533	**<0.001**	0.016	0.911
	post-T	-	-	0.108	0.497	-	-	−0.260	0.097	-	-		-	-	-	0.109	0.452		-	0.323	0.022	-	-	0.450	**0.001**
**Leukocyte activation CD49e EVs**	pre-T	0.163	0.307	0.051	0.750	0.113	0.484	−0.138	0.382	0.422	**0.002**	−0.015	0.915	-	-	0.409	**0.003**	0.648	**<0.001**	0.237	0.097	0.795	**<0.001**	0.243	0.090
	post-T	-	-	0.239	0.127	-	-	−0.188	0.233	-	-	0.109	0.452	-	-	-		-	-	0.490	<0.001	-	-	0.617	**<0.001**
**Platelets CD41b EVs**	pre-T	0.199	0.211	0.013	0.936	0.294	0.062	−0.098	0.536	0.442	**0.001**	0.292	**0.039**	0.648	**<0.001**	0.256	0.073	-	-	0.321	0.023	0.735	**<0.001**	0.358	**0.011**
	post-T	-	-	0.243	0.121	-	-	0.098	0.538	-	-	0.323	**0.022**	-	-	0.490	**<0.001**	-	-	-		-	-	0.686	**<0.001**
**Endothelial cells CD146 EVs**	pre-T	0.075	0.643	0.149	0.347	0.134	0.403	0.051	0.750	0.533	**<0.001**	0.119	0.411	0.795	**<0.001**	0.274	0.054	0.735	**<0.001**	0.340	0.016	-	-	0.379	**0.007**
	post-T	-	-	0.130	0.412	-	-	−0.151	0.339	-	-	0.450	**0.001**	-	-	0.617	**<0.001**	-	-	0.686	<0.001	-	-	-	-
**B cells CD20 EVs**	pre-T	0.398	**0.010**	0.113	0.478	0.332	**0.034**	−0.008	0.960	0.683	**<0.001**	0.272	0.056	0.394	**0.005**	0.052	0.722	0.517	**<0.001**	0.200	0.163	0.519	**<0.001**	0.089	0.539
	post-T	-	-	−0.004	0.980	-	-	−0.010	0.950	-	-	0.479	**<0.001**	-	-	0.016	0.910	-	-	0.386	0.006	-	-	0.437	**0.002**

**Table 6:** The correlation analyses between the parameters of the immune system assessed using extracellular vesicles (EVs), with the tumor size, tumor fraction, administered activity dose (MBq), the interval until death (days), and 60-day survival, were performed by determining the Spearman correlation significance and Spearman r. Expression levels of cluster of differentiation (CD) proteins on the surface of EVs isolated from hepatocellular carcinoma patients before (pre-T) and after selective internal radioembolization treatment (post-T). A p value below 0.05 was considered statistically significant. Abbreviations: IL, interleukin.

Parameter		Tumor Size (cm^3^)		Tumor Fraction (%)		Administered Activity Dose (MBq)		Interval Until Death (Days)		60-Day Survival	
Immune Parameters (All TP)	Time Point	r	*p* Value	r	*p* Value	r	*p* Value	r	*p* Value	r	*p* Value
**Leukocytes (Gpt/L)**	pre-T	0.365	**0.012**	0.339	**0.046**	0.564	**<0.001**	0.267	0.207	−0.438	**0.001**
	post-T	0.123	0.416	0.099	0.571	0.530	**<0.001**	0.087	0.685	−0.307	**0.032**
**Thrombocytes (Gpt/L)**	pre-T	0.442	**0.002**	0.421	**0.012**	0.577	**<0.001**	0.210	0.326	−0.263	0.065
	post-T	0.408	**0.005**	0.417	**0.013**	0.597	**<0.001**	0.331	0.114	−0.054	0.712
**IL-6 (pg/mL)**	pre-T	0.061	0.709	0.069	0.727	−0.257	0.105	−0.475	0.054	−0.234	0.141
	post-T	−0.100	0.533	−0.199	0.300	−0.113	0.476	0.077	0.760	−0.223	0.156
**IL-8 (pg/mL)**	pre-T	0.307	0.054	0.408	**0.031**	0.277	0.079	−0.390	0.122	−0.443	**0.004**
	post-T	0.189	0.237	0.339	0.072	0.122	0.440	−0.534	**0.023**	−0.378	**0.013**
**Leukocyte activation CD45 EVs**	pre-T	−0.087	0.561	−0.044	0.801	−0.184	0.205	−0.280	0.185	−0.287	**0.043**
	post-T	0.139	0.352	0.140	0.422	−0.040	0.785	0.016	0.942	0.031	0.833
**Leukocyte activation CD49e EVs**	pre-T	0.163	0.274	0.117	0.504	−0.009	0.952	−0.070	0.744	−0.341	**0.015**
	post-T	−0.114	0.444	−0.146	0.404	0.063	0.666	0.364	0.080	−0.0034	0.813
**Platelets CD41b EVs**	pre-T	0.140	0.625	0.154	0.349	−0.005	0.377	0.039	0.856	−0.305	**0.031**
	post-T	−0.018	0.907	−0.173	0.321	0.034	0.818	−0.069	0.750	−0.168	0.244
**Endothelial cells CD146 EVs**	pre-T	0.216	0.348	0.201	0.144	−0.029	0.247	−0.129	0.549	−0.413	**0.003**
	post-T	0.213	0.151	0.029	0.869	0.170	0.242	0.295	0.162	0.016	0.911
**B cells CD20 EVs**	pre-T	−0.062	0.464	−0.102	0.681	−0.087	0.560	−0.251	0.236	−0.449	**0.001**
	post-T	0.195	0.188	0.019	0.912	0.079	0.589	0.112	0.602	−0.168	0.244

## Data Availability

The data can be obtained, upon reasonable request, from the corresponding author.
